# Quantitative clinical and autoimmune assessments in stiff person syndrome: evidence for a progressive disorder

**DOI:** 10.1186/s12883-018-1232-z

**Published:** 2019-01-03

**Authors:** Goran Rakocevic, Harry Alexopoulos, Marinos C. Dalakas

**Affiliations:** 1 Department of Neurology, Thomas Jefferson University, Philadelphia, USA; 2 Neuroimmunology Unit, Department of Pathophysiology, Faculty of Medicine, National and Kapodistrian University of Athens, Athens, Greece

**Keywords:** Stiff person syndrome, GAD, Autoimmune diseases, Inhibitory synapses

## Abstract

**Background:**

Stiff Person Syndrome (SPS) is an under-diagnosed disorder that affects mobility and the quality of life of affected patients. The aim of the study is to describe the natural history of SPS, the extent of accumulated disability and the associated clinical and immunological features in patients followed for up to 8 years in a single center.

**Methods:**

Our collective cohort included 57 SPS patients. Additionally, 32 of these patients were examined every 6 months for a two-year period in a longitudinal study protocol, to assess disease progression using quantitative measures of stiffness and heightened sensitivity.

**Results:**

The most frequent initial symptom was leg stiffness, followed by paraspinal muscle rigidity and painful spasms in 95% of the patients. Although none of the patients required assistance for ambulation during the first 2 years of disease onset, 46 patients (80%) lost the ability to walk independently during our follow-up, despite symptomatic medications. In the longitudinal cohort, the number of stiff areas increased (*p* < 0.0001), consistent with worsening functional status and quality of life. High-titer anti-GAD antibodies were present in serum and CSF with elevated intrathecal GAD-specific IgG synthesis, but they did not correlate with clinical severity or progression.

**Conclusions:**

This large study on SPS patients, combining an eight-year follow-up at a single center by the same leading neurologist and his team, is the first to provide longitudinal data in a large patient subgroup using objective clinical measures. One of the main findings is that SPS is a progressive disease leading to physical disability over time.

## Background

Stiff Person Syndrome (SPS) is characterized by rigidity of the truncal muscles with superimposed episodic and often painful muscle spasms, heightened sensitivity to external stimuli, particularly tactile and auditory, and high-titer anti-GAD antibodies [1–6]. Co-contracture of agonist and antagonist muscles and continuous involuntary firing of motor units at rest are the cardinal pathophysiological hallmarks of the disease [7, 8]. The patients also demonstrate marked anticipatory anxiety and task-specific phobias due to unexpected startle responses. SPS is often associated with other autoimmune conditions, most commonly Type I diabetes, which is also an anti-GAD associated disease [9, 10].

Several clinical variants have been described including: ‘stiff-limb syndrome’, a limited form that spares the trunk [11, 12]; a cerebellar variant (SPS-Cer) where cerebellar symptoms are superimposed on the stiffness resulting in prominent gait ataxia [4], a paraneoplastic variant mostly associated with antibodies against amphiphysin or gephyrin [13, 14]; SPS with myoclonus (‘jerking-man syndrome’), associated with antibodies to glycine receptor, now synonymous with Progressive Encephalomyelitis with Rigidity and Myoclonus; and SPS with epilepsy and dystonia [15–22].

The symptoms are fluctuating and it is unclear whether SPS patients accumulate disability over time. We describe a series of patients, evaluated for up to 8 years in a single center with clinical, immunological, imaging and neurophysiological measures and provide prospective longitudinal data, in a subgroup of patients, on disease progression using validated scales that quantified stiffness and spasms.

## Methods

### Patient recruitment and follow-up assessments

Fifty seven SPS patients were recruited (1999–2006) at the Neuromuscular Diseases Section of the National Institutes of Health (NIH) after signing informed consent. All patients were evaluated by the same senior clinician (MCD) and his fellows (mostly GR who completed the entire longitudinal study) and fulfilled the following criteria [4, 6]: 1) insidious onset of rigidity in axial muscles; 2) continuous co-contraction of agonist and antagonist muscles with inability to relax, as confirmed by electrophysiology; 3) episodic spasms often triggered by unexpected external stimuli or emotional upset; and 4) no other neurological diseases that could account for stiffness. Electromyography was assessed in all the patients either from patient records (*n* = 21) or performed by us (*n* = 36). Brain MRI was performed in 44 patients and was selectively repeated during the follow-up period to exclude other processes or search for cerebellar atrophy in those with concurrent cerebellar ataxia.

During the follow-up visits, all disease-related factors were recorded, including frequency and condition of falls, ability to work or carry out daily activities, concurrent medical and psychiatric illnesses, other neurological or autoimmune diseases and response to medications.

Thirty-two patients were enrolled in a distinct longitudinal study protocol, where Stiffness and Heightened Sensitivity scores were systematically recorded using previously validated scales [23], every 6 months for a two-year period, to assess disease progression. During this period, the patients remained on their minimum tolerated doses of symptomatic therapies including various muscle relaxants and anti-anxiety drugs, but without immunotherapy at the discretion of their treating neurologists. No precipitous worsening of SPS symptoms occurred during this 2-year follow-up in any of above patients; hence, no rescue immunotherapies were administered. After this period, they were offered participation in a rituximab trial or were treated with IVIg.

### Neuropsychiatric symptomatology

All patients were assessed for neuropsychiatric symptoms through structured interviews. The validated Structured Interview for the Diagnostic and Statistical Manual for Mental Disorders (DSM-IV) Axis I (SCID-I/P) was additionally administered to 10 consecutive patients to explore the origin of anxiety and frequency of phobic or other psychiatric disorders and assess their personality characteristics and cognitive functioning, as described [24].

### Immunogenetics

Immunogenetic associations were investigated by HLA-I and II allele-specific oligonucleotide typing in genomic DNA extracted from peripheral blood of 34 patients and compared to healthy controls from the NIH database matched for age, ethnicity, and Type-I diabetes.

### Immunological studies

The following autoantibodies were tested: anti-thyroid, anti-parietal cell, anti-gliadin, intrinsic factor blocking antibody, anti-islet cell, ANA, anti-cardiolipin, anti-RNP, anti-SSA/SSB, anti-SM, anti-striated, anti-smooth muscle, anti-microsomal, anti-mitochondrial, anti-acetylcholine receptor, anti-GM1, hepatitis B/C, HIV, HTLV-I and CMV. Immunoglobulin and complement levels were also tested.

Anti-GAD antibodies were initially tested in all patients and serially thereafter, in the 32 patients enrolled in the longitudinal protocol, by radioimmunoassay (RIA) using a commercial kit (Kronus, ID). Lumbar puncture was performed in 48 patients but CSF could not be obtained from 5 due to severe lumbar stiffness. CSF was examined for GAD antibodies, total immunoglobulins, oligoclonal bands, IgG index and intrathecal GAD-specific IgG synthesis, as previously described [6, 25, 26]. Anti-GAD antibodies were also measured in serial CSF’s (first and 2-year visits) in 10 patients by ELISA (Euroimmune, Lubeck). In 50 patients, sera were also tested for anti-glycine receptor and anti-GABAA antibodies using in-house cell-based immunofluorescent assays on live cells (cDNA clones kindly provided by Prof. A. Vincent, Oxford and Prof. J. Dalmau, Barcelona), as described [27].

## Results

Among the 57 patients, 39 were women and 18 men (ratio 2:1); 46 were Caucasians and 11 African Americans. The mean age of disease onset was 42 years (range, 22–60) and the mean disease duration at enrollment 11 years (range, 3–32). The duration between symptom onset and time of diagnosis was 5 years (range: 1–19).

### Clinical presentation at onset

The most common initial symptom was insidious onset of proximal leg stiffness, reported by 19 patients (33%). The second most common symptom was rigidity in the lumbosacral paraspinal muscles (16 patients, 28%), followed by rigidity in the thoracic and abdominal muscles (6 patients, 10%). When first examined, axial muscle stiffness (truncal and proximal legs), lumbar hyperlordosis and impaired gait were detected in 39 patients (68%); sixteen of them (28%) had also various degrees of facial muscle stiffness (Table 1). Muscle pain related to stiffness and spasms was reported by all but two patients. Six patients (10%) had asymmetric presentation in one limb (‘stiff-leg syndrome’), and two others also had severe abnormal hand posturing (pseudo-dystonia). Isolated rigidity of distal leg muscles resembling dystonia was the first symptom in 3 patients. Prominent stiffness only in cervical paraspinal muscles was the initial presentation in one patient; in another, SPS was preceded by prominent oscillopsia, opsoclonus and nystagmus.

**Table 1:** Predominant distribution of stiffness and other neurological signs in 57 patients with Stiff Person Syndrome

Symptoms	n (%)
*Increased tone in:*	
Face	16 (28)
Cervical paraspinal and shoulder girdle muscles	11 (19)
Thoracic paraspinal and abdominal wall muscles	14 (24)
Lumbosacral paraspinal muscles	16 (28)
Proximal leg muscles	17 (30)
Combined lumbosacral paraspinal and proximal leg muscles	33 (58)
Axial (diffuse paraspinal and proximal limb muscles)	39 (68)
Asymmetry with one limb predominant (stiff-limb)	6 (10)
Distal limb stiffness	4 (7)
*Spasms*	50 (88)
*Other associated symptoms:*	
Ocular symptoms	13 (23)
Cerebellar symptoms and signs	7 (12)
*Functional impairment resulting in the following*:	
Stiff or impaired gait	54 (94)
Hyperlordosis	48 (84)
Shortness of breath	11 (19)
Need for cane	26 (45)
Need for walker	14 (24)
Need for wheelchair	9 (15)
Inability to work	48 (84)
Startle response	55 (96)
Anxiety and task-specific phobias	52 (91)
Depressed mood	3 (5)

Cerebellar symptoms including dysmetria, marked gait ataxia or nystagmus, without axial muscle stiffness, were the initial presentation in 5 patients (9%); they too developed typical SPS after a mean period of 5 years (SPS-Cer) with prominent cerebellar symptomatology. All 5 SPS-Cer patients were positive for anti-GAD antibodies.

### Neuropsychiatric symptoms at onset

Exaggerated reaction to various external stimuli and ‘startle response’ was present in all patients except 2 without episodes of muscle spasms. Marked anxiety related to unprotected falls or in anticipation of physically challenging situations was reported in 52 of 57 patients (Table 1). Three patients had refractory depression that coincided with the onset of SPS, while 21 patients experienced chronic anxiety combined with intermittently depressed mood. Simple phobias, such as fear of walking in open and crowded places, crossing a street or taking escalators were reported by 7 patients. Four patients had task-related phobias, such as fear of public speaking. Formal neuropsychiatric testing in ten consecutive patients however, did not meet DSM-IV criteria for phobic disorder [24]; the patients perceived their fears and anxiety as realistic arising from the possibility of falls caused by SPS.

Several patients had been earlier misdiagnosed with conversion or functional disorder because their falls were attributed to avoidant behavior and heightened mental anticipation. Other common misdiagnoses were spinal disease, dystonia or Parkinsonism.

Three patients used alcohol as muscle relaxant prior to diagnosis and finally became alcohol-dependent; two others used cannabis and stimulants (crystal methamphetamine). Many patients reported muscle pain along with painful spasms and some had been on narcotics.

### Overall disease progression in an 8-year observational period

Several patients exhibited diurnal fluctuations of stiffness and spasms with worsening during periods of physical or emotional stress, cold weather or concurrent infections. The stiffness often spread to the adjacent truncal muscles sparing the distal and facial muscles until late in the disease. Episodes of truncal stiffness were often perceived as ‘freezing attacks’ and the sudden falls (due to painful contractions) as seizures necessitating Emergency Room visits for intravenous muscle relaxants. The number of falls during the ambulatory phase averaged up to four per month.

Although none of the patients required assistance for ambulation during the first 1–2 years of disease onset, 46 (80%) lost the ability to walk independently during the course of the disease, and despite receiving symptomatic therapies 26 of them used a cane, 14 a walker and 9 a wheel-chair (Table 1).

The frequency of painful spasms also progressed over time but varied from twenty per day to a few per year and their duration from minutes to hours (*status spasticus)*, as described [21]. In 11 patients (19%) spasms and stiffness also affected the thoracic paraspinal and intercostal or diaphragmatic muscles resulting in respiratory distress. The majority (87%) reported episodic spasms in the areas afflicted by the stiffness; the remaining, either had mild disease or were responding well to symptomatic therapy.

Visual complaints were common. Eleven patients reported double or blurred vision and those with cerebellar ataxia prominent oculomotor dysfunction with nystagmus and oscillopsia [26].

Symptom progression had finally exerted a significant impact on the patients’ quality of life, independence and ability to work. Although at disease onset only two patients quit their jobs due to falls and inability to drive, after a mean period of 4 years only 11 of 57 patients were able to work full or part-time; the majority were on disability benefits.

During the study period, two patients died; one at age 42 from ruptured aneurysm, and another at age 55 due to cardiac arrest of unclear etiology.

### Disease progression with quantitative measures in 32 patients involved in a longitudinal 2-year study period

**Fig. 1:**
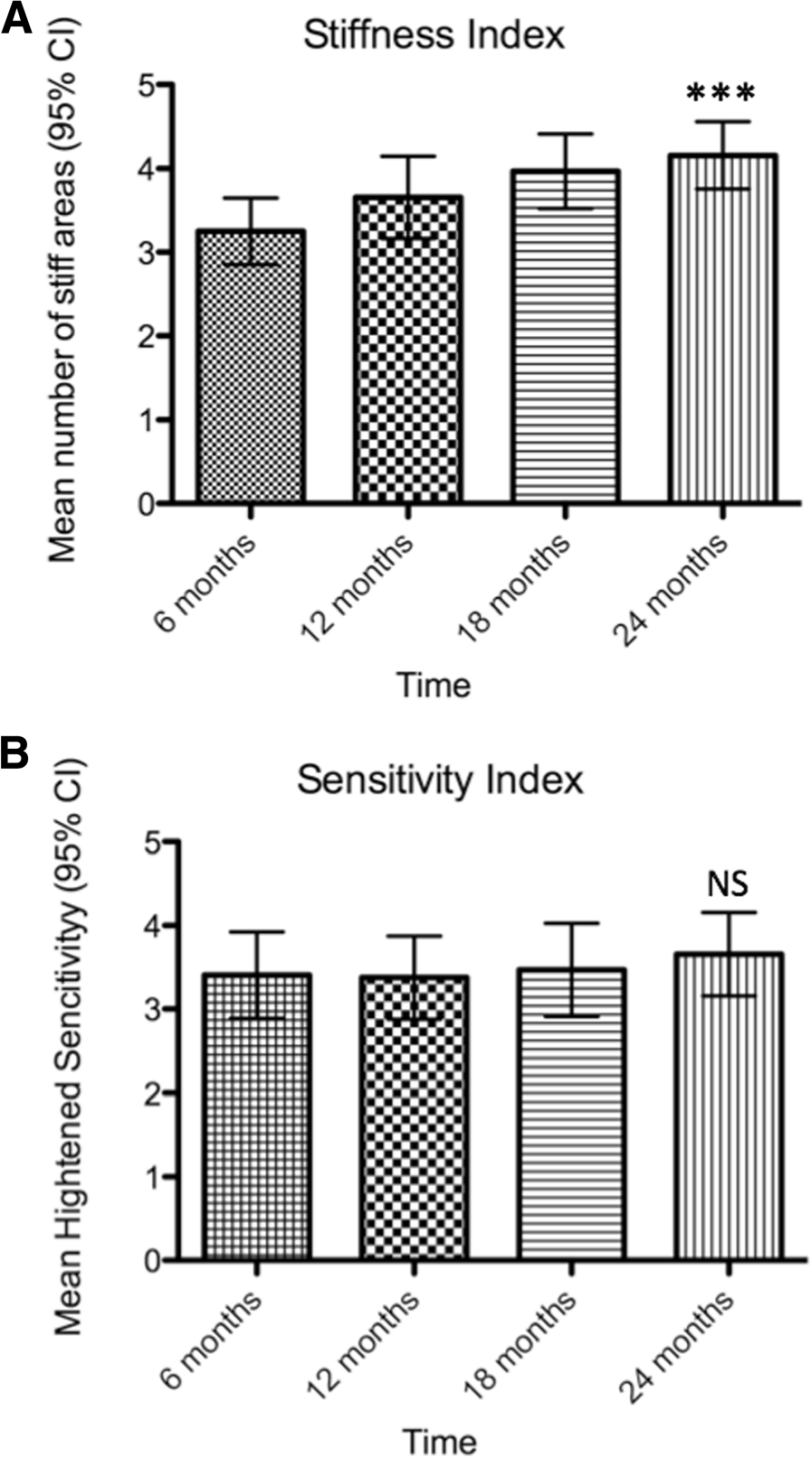
Analysis of stiffness and heightened sensitivity indices for the 32 serially analyzed patients over a 2-year course. **a**: Mean number of stiff areas at 6 months intervals. There is a statistically significant increase (*p* < 0.0001, Friedman test – One way ANOVA) in the number of stiff areas (3.25 at 6 months rising to 4.15 at 24 months) during the observation period. **b**: Mean number of heightened sensitivity scores calculated at 6 months intervals. The observed increase (3.40 vs 3.65) is not statistically significant (*p* < 0.1195, Friedman test – One way ANOVA)

These patients’ progression was measured systematically every 6 months using the Stiffness Index (maximum 6) and Heightened Sensitivity scales (maximum 7). The mean number of stiff areas at entry was 3.25 and the mean heightened sensitivity score 3.40. Two years later, without immunotherapy, the mean number of stiff areas had increased to 4.15 (*p* < 0.0001) and the heightened sensitivity to 3.65 (*p* < 0.5) (Fig. 1a, b). No changes in numbers of affected areas were captured in 9 patients, although their symptom severity increased as reflected by the higher frequency of falls and impaired ability to walk independently or perform their work duties. After the two-year follow up period, five patients could no longer function only with symptomatic therapy and requested intermittent IVIg infusions with considerable benefit. One GAD-positive patient developed metastatic lung adenocarcinoma on study completion; her SPS improved dramatically after chemotherapy.

### Associated diseases at onset and follow-up

Seventeen of 57 patients (30%) developed autoimmune thyroiditis (Table 2); eight became hypothyroid after surgical or radiation treatment and six were on supplemental therapy. Sixteen patients (28%) had Type-1 diabetes diagnosed within 1–3 years from onset of SPS. Eleven patients (19%) had pernicious anemia, two vitiligo, one rheumatoid arthritis and one IgGκ MGUS. Nine patients (15%) had partial complex seizures which were focal or infrequent and well controlled with anti-epileptics. Four GAD-positive patients, without amphiphysin antibodies, developed cancer (breast, prostate, skin, lung adenocarcinoma) several years after onset of SPS but all survived cancer treatment. Two patients had benign tumors, one choroid plexus papilloma and another acoustic neuroma.

**Table 2:** Associated diseases and autoantibodies in SPS patients

Associated diseases	n (%)
Autoimmune thyroiditis	17 (30)
Insulin dependant diabetes mellitus (IDDM)	16 (28)
Pernicious anemia (PA)	11 (19)
Epilepsy	9 (15)
Vitiligo	2 (3)
Rheumatoid arthritis	1 (1)
Autoimmune pancreatitis	1 (1)
Irritable bowl syndrome	1 (1)
Monoclonal gammopathy of unknown significance	1 (1)
Antibodies	n (%)
Anti-nuclear antigen (ANA)	26 (45)
Anti-cardiolipin IgG and IgM (ACA)	10 (17)
(Anti-nuclear and cardiolipin antibodies together)	7 (12)
Thyroid peroxidase antibody	23 (40)
Anti-thyroglobulin antibody	20 (35)
(Thyroid peroxidase and thyroglobulin antibodies together)	16 (28)
Anti-thyroid microsomal antibody	1 (1)
Anti-parietal cell antibody	18 (31)
Anti-islet cell antibody	6 (10)
Anti-gliadin antibody	6 (10)
Intrinsic factor blocking antibody	3 (5)
Anti-JO-1 antibody	3 (5)
Anti-ENA (RNPSM)	2 (3)
Rheumatoid factor (RF)	1 (1)

### Autoantibodies in serum and CSF: Serial studies

All patients had high-titer anti-GAD antibodies (mean 950 nmol/L, normal < 0.02, range: 0.43–11,000), but their titers tested over time varied even in the same patient. There was no correlation between antibody titers and clinical severity or fluctuation as assessed by Stiffness Index and Heightened Sensitivity scores [6, 25]. Eight of 50 (16%) patients harbored anti-glycine receptor antibodies [27]; none of them had anti-GABAA receptor or paraneoplastic antibodies. Other organ-specific autoantibodies were however detected (Table 2).

**Fig. 2:**
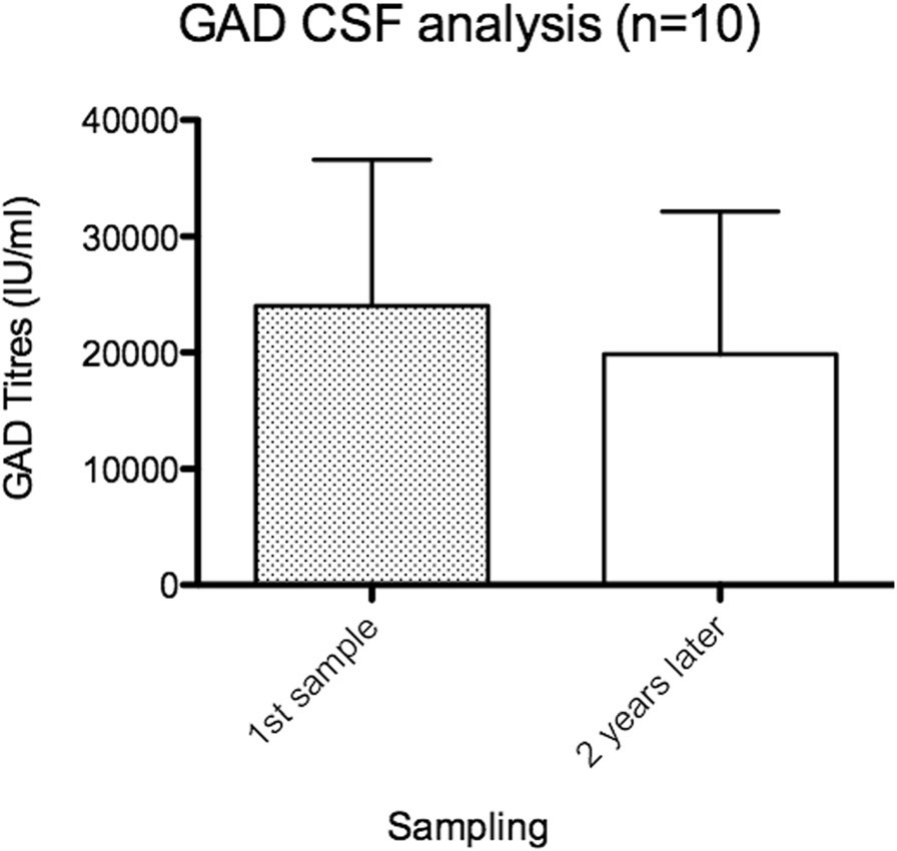
Comparison of CSF anti-GAD antibody titers within a 2-year period. While there is a titer drop in the 2 year-period (mean 24,013 IU/ml vs 19,855 IU/ml) this difference is not statistically significant (*p* = 0,55 two-tailed paired t-test)

In the 32 longitudinally studied patients the anti-GAD serum titers (expressed in Standard Units) ranged from 9000 to 62,000 (mean: 24,000; normal < 1.5) and their CSF from 30 to 2000 (mean: 280; normal: 0). The mean titer ratio of *anti-GAD CSF/ serum* was 0.01 (range, 0.004–0.024) and the mean ratio of *CSF/ serum IgG* was 0.002 (range, 0.001–0.005) indicating a 4-fold increase of specific intrathecal GAD-IgG production. In 10 patients, CSF was also repeatedly tested in a two-year period. The mean titer after 2 years was lower (24,013 International Units/ml vs 19,855 IU/ml) but this difference was not statistically significant (two-tailed, paired t-test, *p* = 0.55) (Fig. 2). Oligoclonal IgG bands were noted in 16 (28%) patients with elevated IgG index in four.

In five SPS patients with cerebellar disease (SPS-Cer), the mean ratio of *anti-GAD CSF/ serum* was 0.03 (range, 0.007–0.057) and the mean ratio of *CSF/ serum IgG* was 0.002 (range, 0.001–0.005), indicating a 10-fold increase of intrathecal antibody production, 2.5-fold higher compared to typical SPS [26], suggesting an enhanced B-cell activation within the CNS, consistent with the more extensive neurological phenotype.

Several patients with prominent muscle rigidity and attacks of muscle spasms showed persistently elevated creatine kinase up to 1000 U/L, but with normal muscle strength. Muscle biopsies in 2 patients showed mild lymphocytic infiltrates and up-regulation of MHC-I, indicative of an indolent inflammatory autoimmune process also affecting the muscle, as reported [6, 9, 21].

### Electrophysiology and imaging

Electrophysiological work-up showed the typical for SPS continuous firing of normal-appearing motor unit potentials at rest with simultaneous co-activation of motor units in agonist and antagonist muscles in 32 of 36 tested patients. Four patients did not complete the EMG because they could not tolerate the procedure due to stiffness and/or pain. Five mildly affected patients or those responding favorably to symptomatic therapy with GABA-enhancing drugs, exhibited normal electromyography. MRI imaging (44/57 patients) was generally uninformative including the patients with SPS-Cer where no atrophy was observed.

### Immunogenetics

Alleles in the HLA-DR or DQ haplotype region, were found in 33/57 tested patients, confirming previous observations [6, 28, 29]. The most frequent allele was the DRB1* 0301, noted in 14/33 patients (42%) compared to 13% frequency seen in normal Caucasians; none of 4 tested African Americans had this allele. A significant association was also noted with the HLA-DQB1* 0201 allele seen in 14/33 patients; seven of them also had Type-1 diabetes and four autoimmune thyroiditis. Associations with the DRB* 3*0101 locus was found in 11 patients and with the 3*0202 in 7. Five patients without diabetes carried the DQB1*0602 protective allele; in contrast, none of the 9 tested patients with Type-1 Diabetes carried this allele, suggesting that DQB1*0602 may be associated with a reduced prevalence of diabetes among SPS patients [28].

## Discussion

We present detailed clinical and immunological 8-year follow-up data from SPS patients examined in a single center by the same leading clinician along with his team. Most importantly, we present 2-year longitudinal data in 32 patients, without immunotherapy, which in spite of variability in disease severity within the same patient, demonstrate that SPS is a progressive disease that leads to physical and social disability. The findings are important in the design of future therapies.

The delay in establishing diagnosis and initiating treatment ranged from 1 to 19 years (mean, 5 years), confirming that SPS still remains a largely under-recognized entity. Symptoms usually started subtly in patients below the age of 50 and progressed slowly thereafter to permanent stiffness or impaired gait; in 15 patients with disease onset > 50 however disability developed much sooner. At first evaluation, the majority of patients (68%) already had diffuse axial muscle stiffness with spasms, hyperlordosis and impaired gait. 23% of patients who started asymmetrically (‘stiff-limb syndrome’) or with mild gait difficulties, ocular disturbances and cerebellar symptomatology, eventually developed the typical SPS phenotype. Anxiety alone or combined with task-specific phobias and depressive mood were eventually universally present resulting in severe social disability [24].

The main novelty of our study is the longitudinally obtained quantitative clinical data from 32 patients which unequivocally demonstrate that SPS is a progressive disease. This was complemented by increasing frequency of falls, need for assistance in walking and daily activities and progressively impaired ability to work in 84% of these patients over the 2-year period. In spite of fluctuations, the overall mean number of stiff areas increased from 3.25 to 4.15 (Fig. 1a); this combined with more frequent spasms, signifies that without immunotherapy SPS is a steadily progressive disease that leads to disability. The heightened sensitivity scores, based on a more variable scale, remained fairly constant during the two-year period (Fig. 1b).

The autoimmune nature of SPS was re-confirmed in our series by the: a) presence of high-titer antibodies against GAD in serum and CSF; b) glycine receptor antibodies present in a patient subset; c) frequent association with other autoimmune diseases and autoantibodies, including thyroiditis and Type-1 diabetes; and d) a strong immunogenetic association with DRB1*0301 and DQB1*0201 haplotypes. Serum and CSF anti-GAD titers, including serial 2-year assessment, did not correlate with clinical severity confirming prior observations in smaller cohorts [4, 6, 9, 21, 25].

The pathogenetic potential of anti-GAD antibodies remains unsettled. The high rate of intrathecal synthesis of GAD-specific IgG signifies B-cell in-situ stimulation in the CSF compartment and possibly in-situ action of antibodies within the CNS, but it is unclear what drives their CNS persistence and why rituximab is ineffective [30]. The reason for the apparent clinical heterogeneity among SPS patients is also uncertain. It was thought to be related to the variable susceptibility of GABAergic neurons to anti-GAD or other still unidentified autoantibodies [31–33] but recent data from two independent studies indicate that all anti-GAD antibodies from SPS or other hyperexcitability syndromes recognize the same dominant GAD epitope [34, 35].

A wide range of medications were used in our series for symptomatic relief, most commonly GABA-enhancing drugs such as sedative-anxiolytics, anti-spasticity and anti-epileptic drugs. Among immunotherapies, high-dose IVIg, as confirmed in a controlled study [23] was effective in a number of patients. It is however puzzling why high-doses of corticosteroids and common immunosuppressants including the anti-B cell agent rituximab [30], which is successfully used in a number of autoimmune neurological diseases [36], have been rather disappointing in SPS except of rare cases responding dramatically to rituximab.

## Conclusions

The progressive and disabling nature of SPS as confirmed in this series [37] indicates the need for prompt initiation of more effective therapies. In particular, proven-to-work immunotherapies such as IVIg might be initiated early if there are signs of clinical worsening, given the fact that SPS symptoms are progressive, as our study demonstrated.

## Abbreviations

CSF: Cerebrospinal fluid
GABAaR: Gamma-Aminobutyric acid a receptor
GAD: Glutamic acid decarboxylase
IVIg: Intravenous immunoglobulin
SPS: Stiff Person Syndrome
